# High-resolution analysis of long-term serum antibodies in humans following convalescence of SARS-CoV-2 infection

**DOI:** 10.1038/s41598-022-12032-8

**Published:** 2022-05-31

**Authors:** Antonio Facciuolo, Erin Scruten, Sean Lipsit, Amanda Lang, Zoë Parker Cates, Jocelyne M. Lew, Darryl Falzarano, Volker Gerdts, Anthony J. Kusalik, Scott Napper

**Affiliations:** 1 Vaccine and Infectious Disease Organization (VIDO), University of Saskatchewan, Saskatoon, SK Canada; 2 Department of Biochemistry, Microbiology, and Immunology, University of Saskatchewan, Saskatoon, SK Canada; 3 Roy Romanow Provincial Laboratory, Saskatchewan Health Authority, Regina, SK Canada; 4 Department of Computer Science, University of Saskatchewan, Saskatoon, SK Canada; 5 Department of Veterinary Microbiology, University of Saskatchewan, Saskatoon, SK Canada

**Keywords:** SARS-CoV-2, Viral host response, Antibodies

## Abstract

Long-term antibody responses to SARS-CoV-2 have focused on responses to full-length spike protein, specific domains within spike, or nucleoprotein. In this study, we used high-density peptide microarrays representing the complete proteome of SARS-CoV-2 to identify binding sites (epitopes) targeted by antibodies present in the blood of COVID-19 resolved cases at 5 months post-diagnosis. Compared to previous studies that evaluated epitope-specific responses early post-diagnosis (< 60 days), we found that epitope-specific responses to nucleoprotein and spike protein have contracted, and that responses to membrane protein have expanded. Although antibody titers to full-length spike and nucleoprotein remain steady over months, taken together our data suggest that the population of epitope-specific antibodies that contribute to this reactivity is dynamic and evolves over time. Further, the spike epitopes bound by polyclonal antibodies in COVID-19 convalescent serum samples aligned with known target sites that can neutralize viral activity suggesting that the maintenance of these antibodies might provide rapid serological immunity. Finally, the most dominant epitopes for membrane protein and spike showed high diagnostic accuracy providing novel biomarkers to refine blood-based antibody tests. This study provides new insights into the specific regions of SARS-CoV-2 targeted by serum antibodies long after infection.

## Introduction

Severe acute respiratory syndrome coronavirus 2 (SARS-CoV-2) is a highly infectious respiratory pathogen responsible for the COVID-19 (coronavirus disease 2019) pandemic. Antibody responses have been of central importance in multiple avenues in the fight against this emerging pathogen^1^. As with other viruses, antibodies that block (i.e., neutralize) the host–pathogen interaction are an essential component of protective immunity. The predominant target of antibody-mediated immunity is spike glycoprotein, which is a critical virulence determinant that facilitates binding and entry into host cells via the angiotensin-converting enzyme 2 (ACE2) receptor^2^. Antibodies that block this interaction can inhibit viral entry^3^, and are a correlate of protection in non-human primates^4^ and humans^5^. Antibody responses have also served as the primary measure in evaluating the magnitude of naturally acquired^6^ and vaccine-induced immunity^7^. Lastly, antibody responses to immunodominant B cell antigens, such as nucleoprotein and spike glycoprotein, have been exploited for the development of serological-based tests for disease surveillance (Foundation for Innovative New Diagnostics SARS-CoV-2 Diagnostic Pipeline; https://www.finddx.org/covid-19/pipeline/).

To date, humoral immune responses to SARS-CoV-2 have focused on responses to the full-length spike glycoprotein or fragments thereof. The earliest studies primarily focused on the magnitude and duration of antibody responses in relation to disease morbidity^8^. Later studies began to investigate the B cell receptor repertoire at various time-points post-infection revealing a dynamic B cell population that continues to evolve well after resolution of infection^9,10^. Additional studies further characterized the antigenic regions of spike glycoprotein based on the information extracted from the memory B cell receptor repertoire^11,12^. A number of studies have resolved the linear epitopes within SARS-CoV-2 using peptide microarrays^13–17^, phage-display libraries^18^, or peptide pools^19,20^. In those cases, serum IgG antibodies were analyzed at early time-points post-infection/post-onset of symptoms (3–60 days). However, this leaves a significant knowledge gap surrounding the epitope-specific antibody responses circulating in blood following resolution of infection beyond 60 days. With the on-going pandemic, this information is critical in understanding the development and maintenance of natural immunity to SARS-CoV-2. Longitudinal studies thus far have examined either the B cell repertoire^11,12^, or antibody responses to full-length, or fragments of, spike glycoprotein^8,21^. A number of studies have demonstrated that anti-spike IgG levels in blood peak around 30–40 days post-infection^22,23^ followed by a marked, rapid decline and are subsequently maintained at a steady-state for up to 12 months following infection^8,21^.

Longitudinal analysis of the B cell receptor repertoire suggests an expanding population with respect to both epitope diversity and clonotype abundance over time^9,11^. This evolving state implicates similar, dynamic changes in the epitope-specific antibodies circulating in blood—the extent to which these immunological events are interlinked remains unknown. Evidence of epitope-specific antibody responses starting to wane at 30 days post-infection have been reported^16^, while expansion of memory B cell clonotypes (specifically to the receptor-binding domain (RBD) and critical neutralizing epitopes)^9,11^ have been described. Thus, it would be of value to identify which epitope-specific antibodies are maintained, and given that neutralizing antibodies persist in blood up to one-year post-infection^8,21^ this could inform which regions to target to neutralize viral activity; such information has potential applications in guiding development of peptide-based vaccines. Given that high anti-spike IgG titers alone do not necessarily equate to neutralizing activity^17^, such analyses could also reveal biomarkers yielding more accurate measures of antibody-based immunity. Moreover, single epitope level analysis might assist in the development of more reliable diagnostic tests given that antibody responses to full-length protein rapidly wane following infection, but the kinetics of this contraction does not affect all epitope-specific antibody responses universally^16^.

In this study, we sought to investigate the epitope-specific serum antibody responses to SARS-CoV-2 that persisted 5 months post-COVID-19 diagnosis (n = 22). Consistent with previous reports, serum spike-specific IgG titers were detected in all COVID-19 convalescent serum samples and contained functional antibodies that demonstrated live virus neutralizing activity or the capacity to block RBD-ACE2 interaction. SARS-CoV-2 epitope-specific IgG responses were determined by comparing the reactivity of COVID-19 convalescent sera to naïve controls (n = 20) using peptide microarrays representing the complete proteome of SARS-CoV-2 and homologs of spike glycoprotein (S), membrane protein (VME1), envelope protein (VEMP), and nucleoprotein (NCAP) from related human coronaviruses (Severe acute respiratory syndrome coronavirus (SARS-CoV), Middle East respiratory syndrome-related coronavirus (MERS-CoV), Human coronavirus-OC43 (HCoV-OC43), and Human coronavirus 229E (HCoV-229E). We discuss how these findings reflect interactions with longer-lived antibody responses that might contribute to long-term protection, reveal more robust serology-based diagnostic markers for disease surveillance, and collectively provide the first single epitope level analysis of the long-term circulating antibody repertoire following SARS-CoV-2 infection.

## Results

### Long-term polyclonal antibodies in COVID-19 convalescent patients can neutralize viral activity

For this study, COVID-19 convalescent sera was obtained from RT-PCR positive cases diagnosed in March 2020 (male, n = 10; female, n = 12; age range 15–79), and blood collected in July/August of 2020 (Supplementary Table S1). For naïve sera, we recruited 37 healthy individuals and 20 samples from this cohort were randomly selected for further analysis (Supplementary Table S2) ensuring equal representation of age and sex (male, n = 10; female, n = 10; age range 20–69).

Spike glycoprotein S1 domain-specific IgG titers were measured for COVID-19 convalescent sera collected at 5 months post-diagnosis (PD), and for naïve sera, by ELISA (S1 and Supplementary Table S2). As expected, S1-specific IgG titers were low in naïve (range 0 to 1/194; median, 1/35; n = 20) and greater in COVID-19 (range 1/417–1/25, 488; median, 1/1417; n = 22) samples. As a surrogate measure for determining functional antibody responses, we measured the capacity of serum antibodies to inhibit recombinant RBD binding to human ACE2 receptor (RBD-ACE2 blocking). Naïve samples displayed low inhibitory activity (range 0.05–13.3%; median, 9.3%), by contrast COVID-19 samples had higher inhibitory activity (range 44–97%; median, 75.1%,). Consistent with previous reports, S1-specific IgG titers highly correlated with RBD-ACE2 blocking (r = 0.89, *p* < 0.001). For COVID-19 samples, micro-neutralization assays were performed using live SARS-CoV-2 virus. All COVID-19 samples, apart from five, had detectable levels of neutralizing antibodies against live virus (range 1/10–1/224; median, 1/20) (Supplementary Table S1). Neutralization titers against live virus showed moderate, but statistically significant, positive correlations to S1-specific IgG titers (r = 0.70, *p* = 0.0003) and RBD-ACE2 blocking (r = 0.71, *p* = 0.0002), consistent with previous analyses^24^. Despite the small sample population in this study, spike-specific IgG titers^23^ and neutralization titers^24^ measured in these convalescent sera collected at 5-month PD are consistent with those previously reported. Although five COVID-19 serum samples had below detectable levels of neutralizing titers against live virus, RBD-ACE2 blocking results demonstrated that functional antibody responses were present in these samples, as such, they were retained in all subsequent analyses.

### Long-term polyclonal antibodies following COVID-19 recognize multiple SARS-CoV-2 epitopes, and cross-react with related human coronaviruses

**Figure 1:**
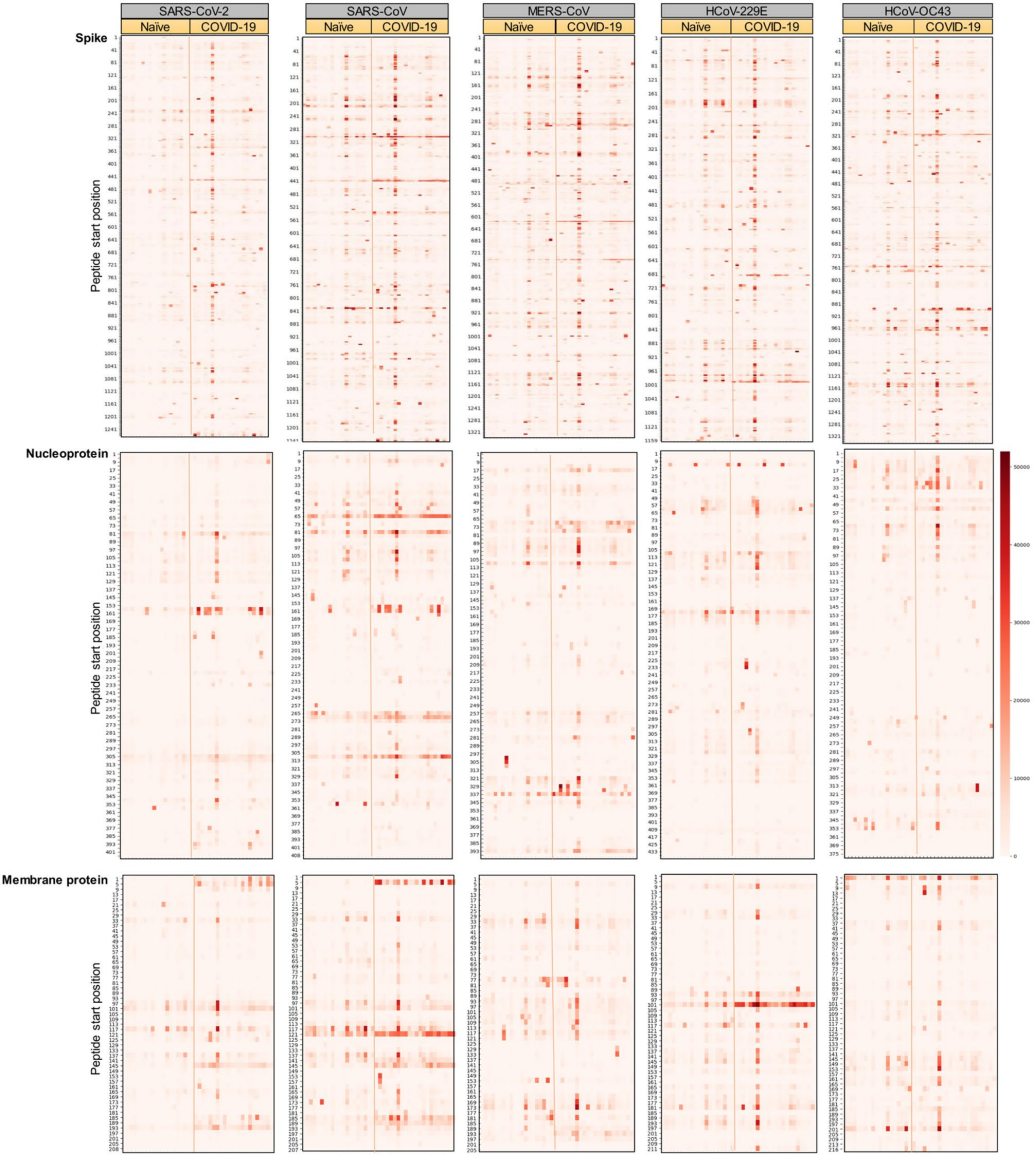
Long-term polyclonal antibodies following COVID-19 recognize multiple SARS-CoV-2 epitopes, and cross-react with related human coronaviruses. Heat-maps displaying the reactivity of COVID-19 convalescent sera collected at 5 months post-diagnosis (n = 22) and naïve sera (n = 20) to individual peptides of spike, nucleoprotein, and membrane protein. For each heat-map, the left and right panels show reactivity with naïve and COVID-19 convalescent sera, respectively. The y-axis represents the linear protein sequence arranged from N-terminus (top) to C-terminus (bottom).

The evaluation of long-term antibody responses following resolution of SARS-CoV-2 infection has primarily focused on responses to either full-length spike protein or the spike RBD domain. Single epitope level analysis of antibody responses, on the other hand, have predominantly been characterized using convalescent sera collected within a few days to at most 60 days post-infection or post-onset of disease. To fill this knowledge gap, we evaluated the specificity of circulating polyclonal antibodies at a later time-point. We performed this analysis on COVID-19 convalescent sera collected at 5 months PD. Each sample was incubated with a pan-coronavirus peptide immunoarray representing the entire proteome of SARS-CoV-2 as consecutive 15-mer peptides with 11 amino acid overlap. Similarly represented on this peptide immunoarray were protein homologs for spike glycoprotein (spike), nucleoprotein (NCAP), membrane protein (VME1), and envelope small membrane protein (VEMP) from related human coronaviruses (SARS-CoV, MERS-CoV, HCoV-OC43 and HCoV-229E). Among naïve and COVID-19 samples, epitope-specific IgG antibody responses against spike, NCAP, and VME1 of SARS-CoV-2 were heterogeneous (Fig. 1); similar individual-level variability was also observed for spike, NCAP, and VME1 belonging to the related human coronaviruses. Analyzing the cumulative signal intensity and distribution of intensities for spike, NCAP, and VME1 peptides revealed that naïve sera samples showed the lowest reactivity towards these peptides from SARS-CoV-2 compared to homologs belonging to SARS-CoV, MERS-CoV, HCoV-229E, and HCoV-OC43 (Supplementary Fig. S1). COVID-19 convalescent sera, when compared to naïve sera, showed the most significant differential change in reactivity against peptides from SARS-CoV-2 spike protein (*p* = 0.047), NCAP of SARS-CoV-2 (*p* = 0.0072) and SARS-CoV (*p* = 0.020), and VME1 of SARS-CoV-2 (*p* = 0.0029) and SARS-CoV (*p* = 0.0072). We further separated spike peptide reactivities for SARS-CoV-2 based on their localization within the S1 and S2 domains. This revealed that IgG responses towards peptides within the S2 domain accounted for more of the differential reactivity (*p* = 0.047) than the S1 domain (*p* = 0.07). We further sought to address whether individual-level reactivities to SARS-CoV-2 spike peptides representing the S1 domain correlated with IgG titers measured against recombinant spike S1 protein by ELISA. There was a moderate, statistically significant positive correlation (r = 0.35, *p* = 0.022) between S1 IgG titers measured by ELISA to cumulative signal intensity of S1 peptides from the peptide immunoarray. Collectively, these data indicated that epitope-specific IgG responses in COVID-19 samples are highly specific to SARS-CoV-2 as reactivity in naïve sera samples is negligible. Further, epitope-specific IgG antibodies to linear spike, NCAP, and VME1 epitopes of SARS-CoV-2 accounted for the greatest differential response in COVID-19 samples. Although IgG antibody responses in COVID-19 samples showed a trend towards elevated reactivity to peptides in related coronaviruses, this cross-reactivity was most evident for SARS-CoV, consistent with previous reports^25^, and to a lesser extent MERS-CoV and HCoV-229E.

### Long-lasting epitope-specific IgG antibodies to SARS-CoV-2 reveal a heterogeneous response with a small subset of shared epitopes

**Figure 2:**
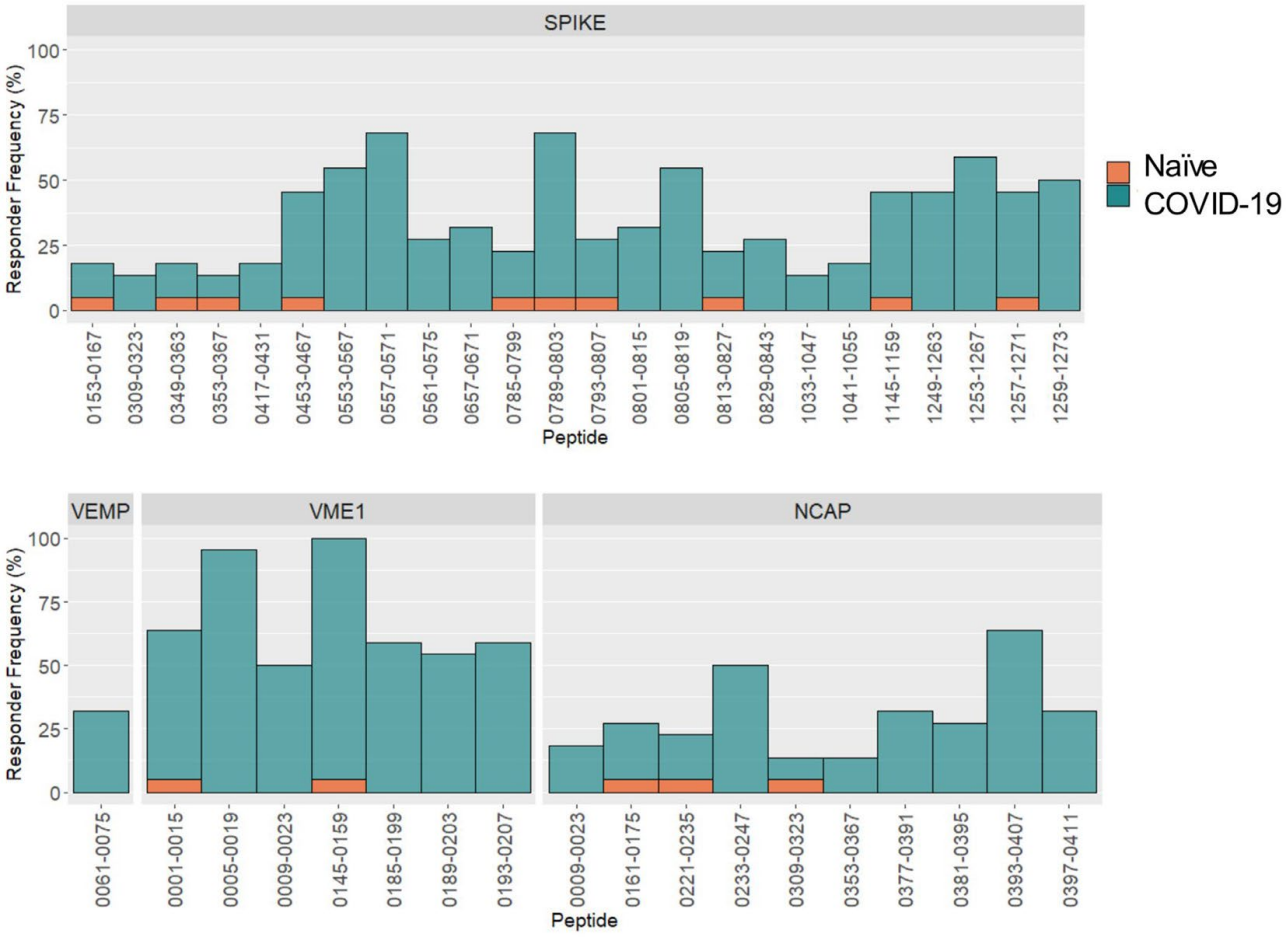
Long-lasting epitope-specific IgG antibodies to SARS-CoV-2 reveal a heterogeneous response with a small subset of shared epitopes. For each differentially reactive peptide (*p*-adjusted value < 0.1), the responder frequency was calculated using a cutoff defined as the mean value of the naïve sera samples plus three standard deviations (n = 20), and percent responders among the COVID-19 convalescent sera (n = 22) collected at 5 months post-diagnosis determined. Only peptides with a responder frequency greater than 10% are displayed. *NCAP* nucleoprotein, *VEMP* envelope small membrane protein, *VME1* membrane protein.

To define differentially reactive peptides (i.e., epitope-specific antibody responses) between naïve (n = 20) and COVID-19 (n = 22) serum samples, we selected for those with a FDR-adjusted *p* value < 0.1 and further filtered to select for those with a responder frequency greater than 10% (Table 1, Fig. 2). The responder frequency cutoff for each peptide was defined as the mean value of the naïve sera samples plus three standard deviations. A total of 129 differentially reactive peptides were identified for SARS-CoV-2 (Table 1, Supplementary Table S3). Their source proteins are, in decreasing order: R1AB (49 peptides), R1A (35 peptides), spike (24 peptides), NCAP (10 peptides), VME1 (7 peptides), VEMP (1 peptide), NS7A (1 peptide), and ORF9B (2 peptides). For SARS-CoV, similar numbers of differentially reactive peptides belonging to spike (19), NCAP (10), and VME1 (5) were identified. By contrast, peptides belonging to spike, NCAP, and VME1 of MERS-CoV, HCoV-OC43 and HCoV-229E yielded few differentially reactive peptides likely owing to the sequence dissimilarity to these species (Table 1). Restricting the analysis of differentially reactive peptides to the most immunodominant (defined in this study as having a responder frequency greater than 45%) revealed the greatest representation of SARS-CoV-2 peptides from spike (10/19) followed by VME1 (7/19) and NCAP (2/19)*.* For these 19 immunodominant epitopes, the signal intensity on the peptide immunoarray with naïve serum samples was below the signal threshold (< 1000 RFU), suggesting these responses are specific to SARS-CoV-2. By contrast, peptides from R1A and R1AB were represented amongst the most differentially reactive and had a high responder frequency; however, many of these peptides bound antibodies in naïve serum samples yielding a high background signal intensity. Although these peptides are discriminatory, they are not specific. For differentially reactive peptides belonging to SARS-CoV-2, 26% of peptides showed a responder frequency greater than 50%, while 66% of the peptides had a responder frequency less than 40%. This suggests that the individual-level epitope responses are greater than population-level epitope responses; this heterogeneity in epitope-specific antibody responses among SARS-CoV-2 infected individuals is consistent with previous single epitope level analyses^13,17,18^.

**Table 1:** Differentially reactive peptides (adjusted *p* < 0.1, responder frequency ≥ 10%) in COVID-19 convalescent sera (n = 22) when compared to naïve sera (n = 20).

	Total	Spike	NCAP	VEMP	VME1	NS7A^a^	ORF9B^a^	R1A^a^	R1AB^a^
SARS-CoV	34	19	10	0	5				
SARS-CoV-2	129	24	10	1	7	1	2	35	49
MERS-CoV	13	5	7	0	1				
HCoV-OC43	11	6	3	1	1				
HCoV-229E	7	4	1	1	1				

*NCAP* nucleoprotein, *VEMP* envelope small membrane protein, *VME1* membrane protein, *NS7A* non-structural protein 7A, *R1A* replicase polyprotein 1a, *R1AB* replicase polyprotein 1ab.

^a^Peptide microarray only contains peptides from SARS-CoV-2.

### Epitope mapping shows distinct clusters of antibody reactivity, and spike epitopes that are both surface-accessible and localize to known neutralizing target sites

**Figure 3:**
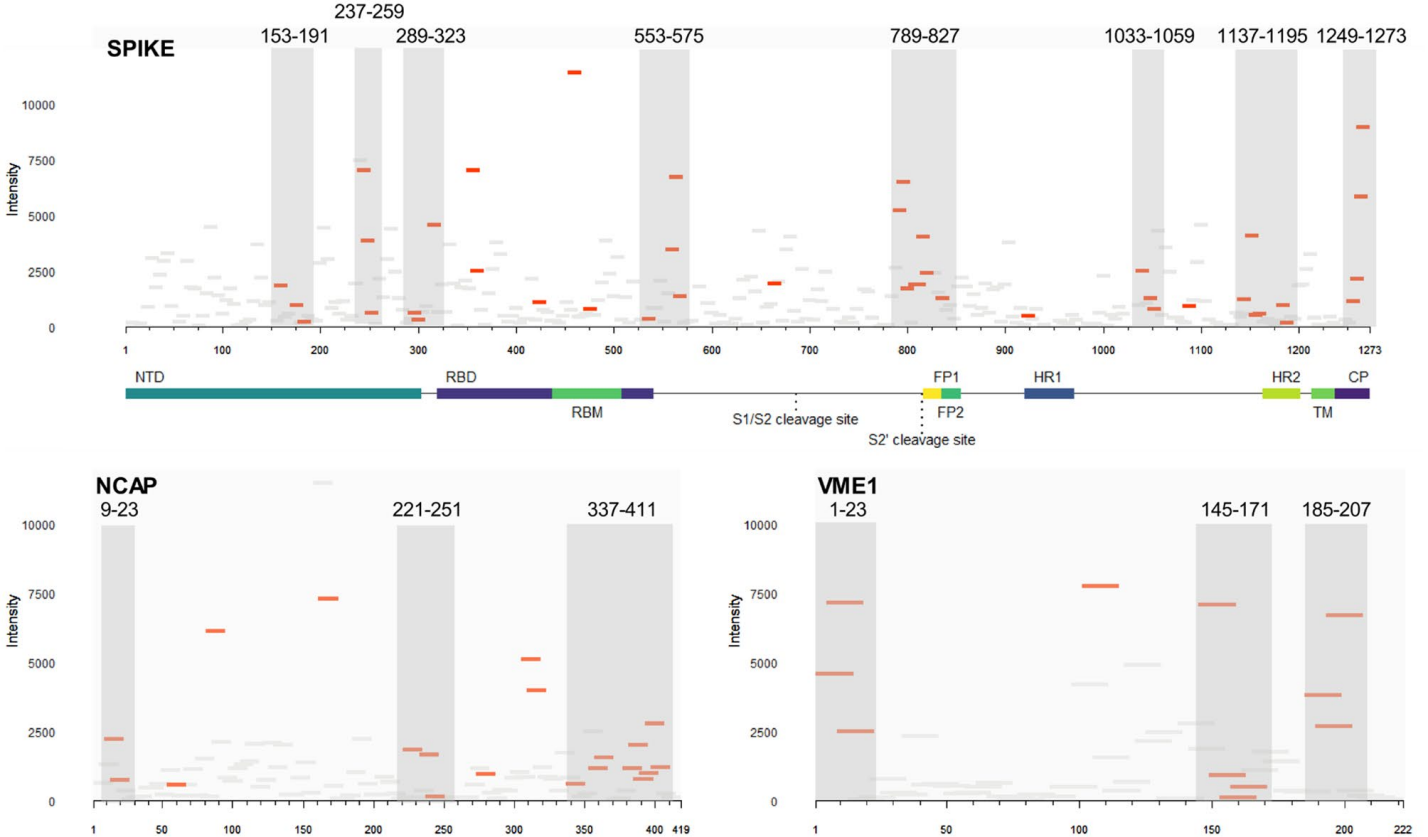
Epitope mapping of SARS-CoV-2 spike, nucleoprotein, and membrane protein reveal distinct clusters of antibody reactivity. Differentially reactive peptides (adjusted *p* < 0.1, red tiles; adjusted *p* > 0.1, grey tiles) mapped on to their respective linear sequence. The x-axis of each panel represents the linear protein sequence arranged N-terminus (left) to C-terminus (right); y-axis represents the mean signal intensity for the COVID-19 convalescent sera (n = 22). Grey boxes demarcate epitope clusters, the sequence position is displayed for each. *NCAP* nucleoprotein, *VME1* membrane protein, *NTD* N-terminal domain, *RBD* receptor-binding domain, *RBM* receptor-binding motif, *FP* fusion peptide, *HR* heptad repeat, *TM* transmembrane domain, *CP* cytoplasmic tail.

**Figure 4:**
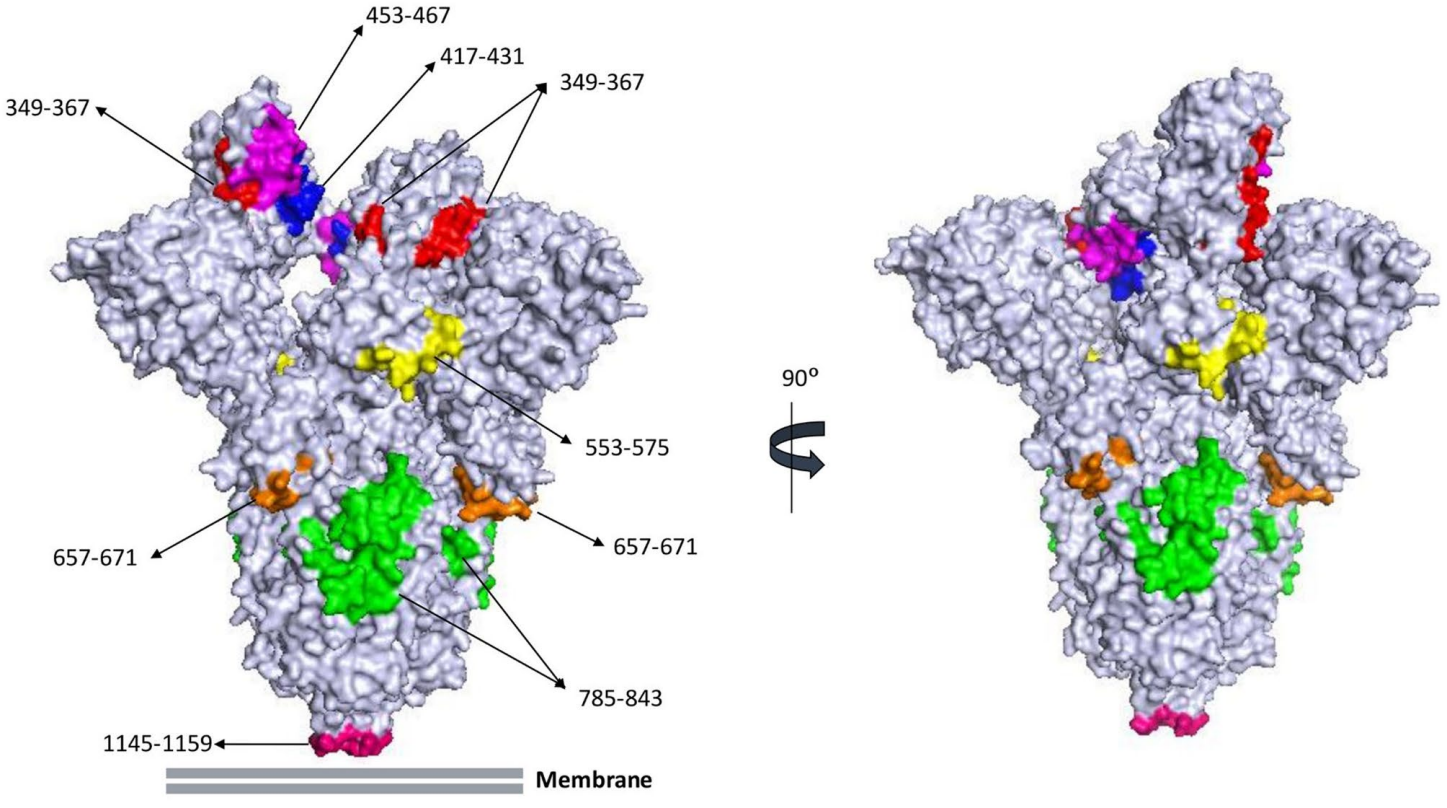
The majority of anti-spike polyclonal antibodies bind to surface-accessible regions, and known neutralizing targets, of the protein. Shown is the molecular simulated 3D structure of trimeric spike glycoprotein, side views, in the open conformation. Epitopes are uniquely colored. Image rendered using PyMOL.

To define the antigenic regions of SARS-CoV-2 spike, NCAP, and VME1 that are bound by IgG antibodies present in COVID-19 convalescent sera at 5 months PD, we mapped each peptide to their respective index sequence (Fig. 3). Within the S1 domain of spike, three epitope clusters were identified in the N-terminal domain, two single epitopes in the N-terminus of RBD (residues 349–367 and 417–431), two single epitopes in the receptor-binding motif (RBM) (residues 453–483 and 469–483), one epitope cluster in the C-terminus of the RBD domain, and one single epitope adjacent to the S1/S2 cleavage site (residues 657–671). In the S2 domain, four clusters are observed, including: one encompassing the S2’ cleavage site, fusion peptide (FP)1, and FP2 domains; one centered on the heptad repeats (HR)2 domain; and one localizing to the cytoplasmic tail. Mapping these epitopes onto the 3D structure of trimeric spike glycoprotein in its open conformation revealed that all but one epitope (residues 1033–1059) contains surface accessible residues (Fig. 4).

For VME1, three linear epitope clusters are identified, and a single epitope is found at residues 101–115 (Fig. 3). For NCAP, a large epitope cluster localizes to the C-terminus of the protein, a centrally located cluster, two small clusters at the N-terminus, and six additional single epitopes were identified (Fig. 3). The most differentially reactive NCAP peptides were single epitopes at positions 81–95, 161–175, and 305–323. Collectively, COVID-19 convalescent sera show greater reactivity in terms of magnitude (Fig. 3) and responder frequency (Fig. 2) with VME1 peptides than NCAP peptides. Taken together, this suggests that spike and VME1 linear epitope-specific responses are immunodominant among the circulating antibody repertoire long-after resolution of infection.

### Immunodominant SARS-CoV-2 epitopes are highly specific and discriminatory, and exhibit minimal cross-reactivity to related human coronaviruses beyond SARS-CoV

**Figure 5:**
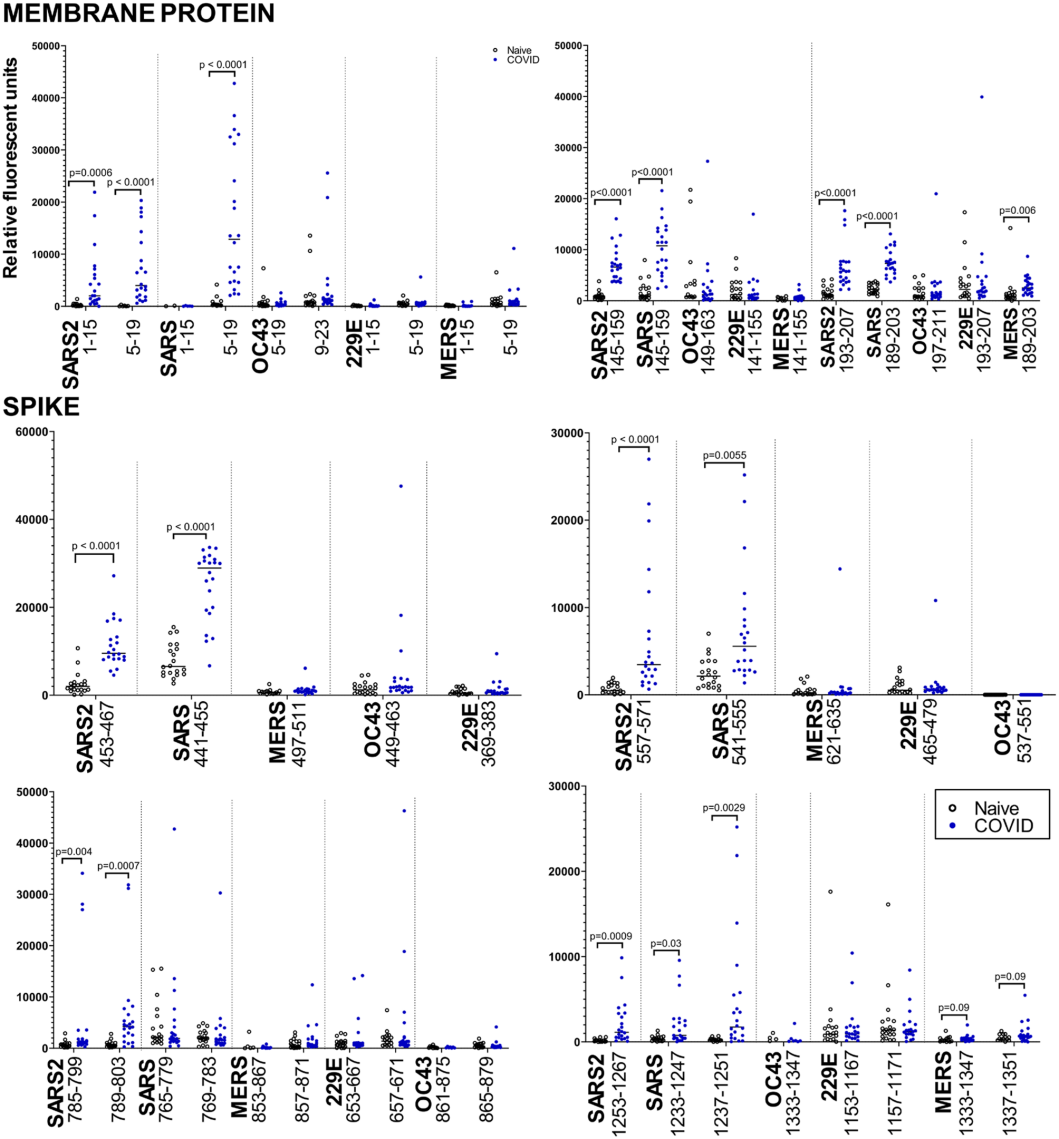
Immunodominant SARS-CoV-2 epitopes are highly specific and discriminatory, and exhibit minimal cross-reactivity to related human coronaviruses beyond SARS-CoV. Data presented for the three immunodominant membrane protein epitopes (peptides 1–19, 145–159, and 193–207), and four spike epitopes (peptides 453–467, 557–571, 785–803, and 1253–1267). Dots represent average signal intensity (relative fluorescent units) for individual samples (open circles, naïve sera, n = 20; blue circles, COVID-19 sera, n = 22). Sequence alignments were used to identify homologous regions in related coronaviruses. P-values were calculated using the Mann–Whitney U test and adjusted for false-discovery using the Benjamini–Hochberg correction. SARS2, SARS-CoV-2; SARS, SARS-CoV; MERS, MERS-CoV; OC43, hCOV-OC43; and 229E, hCOV-229E.

We performed sequence alignment of the full-length proteins to identify regions of similarity. Using these sequence alignments, we first investigated the specificity of the immunodominant NCAP, VME1, and spike peptides by comparing the reactivities to these peptide regions to those from related coronaviruses. For each immunodominant SARS-CoV-2 peptide and its homologs, we plotted the individual reactivities for all sera samples (Fig. 5). Epitope-specific antibody responses to VME1 peptides showed a high responder frequency for peptides 1–15 (63%), 5–19 (95%), 145–159 (100%), and the cluster 185–207 (54–59%). There is a high sequence similarity of the three latter peptide sequences to those in the SARS-CoV proteome resulting in a high degree of cross-reactivity (Fig. 5). The reactivity of COVID-19 convalescent antibodies to the N-terminal peptide of SARS-CoV-2 is highly specific as reactivity to the N-terminus peptide of VME1 SARS-CoV, MERS-CoV, HCoV-OC43, and HCoV-229E showed negligible cross-reactivity. For SARS-CoV-2 VME1 peptides 145–159 and 193–207, homologous peptides from MERS-CoV, HCoV-OC43, and HCoV-229E showed similar levels of reactivity with both naïve and COVID-19 convalescent sera. Unlike the epitope-specific response to the peptides from SARS-CoV-2 or SARS-CoV, antibody responses to these peptides from related coronaviruses cannot discriminate between naïve and COVID-19 convalescent sera. Although VME1 epitope-specific antibody responses are not currently regarded as critical mediators of immunity, these observations suggest the specificity of these peptides could contribute to the development of specific serological-based diagnostic tests for disease surveillance.

SARS-CoV-2 NCAP peptides 233–247 and 393–407 displayed the highest responder frequency (50% and 63%, respectively); the other six differentially reactive NCAP peptides had responder frequencies below 33%. Sequence alignments identified peptides within SARS-CoV (233–247) and MERS-CoV (225–239) that share sequence similarity to peptide 233–247, and a peptide within SARS-CoV (393–407) that shares sequence similarity to peptide 393–407. Only reactivity to SARS-CoV peptide 233–247 could discriminate between naïve and COVID-19 convalescent sera (*p* = 0.04). This suggests that reactivity to these peptides is specific, and cross-reactivity to related coronaviruses is restricted. Compared to the NCAP epitope-specific responses reported at earlier time-points and their reported performance in discriminating naïve from COVID-19 convalescent sera^13,18^ these data suggest linear epitope-specific responses to NCAP, both in terms of magnitude of reactivity and breadth of epitopes recognized, have significantly contracted at 5 months post-diagnosis.

For SARS-CoV-2 spike epitopes, we examined two epitopes within the S1 domain (453–467 and 557–571) and two epitopes in the S2 domain (789–803 and 1253–1267) as they displayed the highest responder frequency and magnitude of change among the differentially reactive spike epitopes. Epitope-specific responses to SARS-CoV-2 peptides 453–467, 557–571, and 1253–1267 also coincided with reactivity to SARS-CoV peptides 441–455, 541–555, and 1233–1251 suggesting the sequence conservation between these peptide sequences enables cross-reactivity (Fig. 5). Linear peptides from related coronaviruses MERS-CoV, HCoV-229E, and HCoV-OC43 similar in sequence to 453–467 and 557–571 SARS-CoV-2 epitopes revealed negligible reactivity with COVID-19 convalescent sera; for epitope 1253–1267, increased cross-reactivity was apparent for a similar peptide within HCoV-229E with both naïve and COVID-19 convalescent sera but was not discriminatory. For SARS-CoV-2 peptide 789–803, two SARS-CoV peptides with shared sequence similarity reacted with both naïve and COVID-19 convalescent sera, but these epitope-specific responses could not discriminate between naïve and COVID-19 convalescent serum samples. Based on the sequence alignments, peptides from MERS-CoV, HCoV-OC43, and HCoV-229E shared little sequence conservation and expectedly showed negligible antibody binding with both naïve and COVID-19 convalescent sera.

### Epitope-specific antibody responses show high diagnostic accuracy and might provide more refined surrogate markers of protection

Given the responder frequency, differential reactivity, and high-specificity of these peptides, we next determined if any of these epitope-specific antibody responses correlate with live virus neutralizing titers. Only spike epitope 557–571 showed a significant (*p* = 0.0014) positive correlation (r = 0.639) with live virus neutralization. This is greater than that observed for S1 IgG titers (r = 0.50) and RBD-ACE2 blocking (r = 0.54) among this sample population. We further determined the diagnostic utility of these immunodominant peptides and compared that to S1 IgG titers measured by ELISA (Supplementary Table S4). All four immunodominant spike epitopes (453–467, 557–571, 789–803, and 1253–1267) and NCAP epitope 393–407 had high AUC values (0.961, 0.946, 0.898, 0.888, and 0.968, respectively) but were not superior to S1 IgG ELISA (AUC 1.0). VME1 epitopes 5–19 and 145–159 had the highest AUC values (1.0) displaying similar diagnostic accuracies as S1 protein. Combining the reactivities of the two VME1 and three spike epitopes (557–571, 789–803, and 1253–1267) showed a high level of specificity and sensitivity [Sp 95% (76–99); Sn 100% (85–100)] and diagnostic accuracy (AUC 1.0) equivalent to S1 protein [Sp 95% (76–99); Sn 100% (85–100)]]. Collectively, these epitope-specific responses might provide novel surrogate markers for disease surveillance and to monitor naturally acquired antibody-mediated immunity.

### SARS-CoV-2 induced antibodies cross-react with epitopes from related human coronaviruses

In the current study, epitope-specific antibody responses to SARS-CoV were significantly elevated in COVID-19 convalescent serum samples relative to naïve sera (Table 1, Supplementary Fig. S1). This observation is consistent with increased cross-reactivity of B cell receptor repertoire to SARS-CoV^10^, and enhanced reactivity of serum antibodies to spike protein of SARS-CoV and MERS-CoV^25^. We next looked at the differentially reactive spike peptides from MERS-CoV, HCoV-OC43, and HCoV-229E to identify any sequence conservation within the SARS-CoV-2 proteome that could explain the enhanced responses observed with these peptides. SARS-CoV-2 spike peptide 813–827 shows high sequence similarity to peptide sequences in SARS-CoV (789–803), MERS-CoV (885–899), HCoV-OC43 (901–915), and HCoV-229E (685–699). Each of these peptides were differentially reactive with COVID-19 convalescent sera. Similarly, SARS-CoV-2 spike peptide 1145–1159 shares sequence similarity with peptides from SARS-CoV (1125–1139), MERS-CoV (1225–1239) and HCoV-OC43 (1229–1243); each of these was differentially reactive with COVID-19 convalescent sera. These highly cross-reactive epitopes (Supplementary Fig. S2) were previously identified by peptide microarray analysis^13^, and phage display assays^18^. The other spike and NCAP peptides from MERS-CoV, HCoV-OC43, and HCoV-229E that were differentially reactive with COVID-19 convalescent sera did not display a clear consensus sequence to SARS-CoV-2 peptides. Although SARS-CoV-2 induced antibodies that cross-react with linear epitopes from related coronaviruses, antibody responses to SARS-CoV peptides displayed similar signal intensities and responder frequencies. Cross-reactivity of SARS-CoV-2 epitope-specific antibodies to related coronaviruses MERS-CoV, HCoV-OC43, and HCoV-229E were much less evident, consistent with that previously reported^13,18^.

### Spike protein immunosignature reveals long-term antibodies bind key regions associated with emerging variants of concern

**Figure 6:**
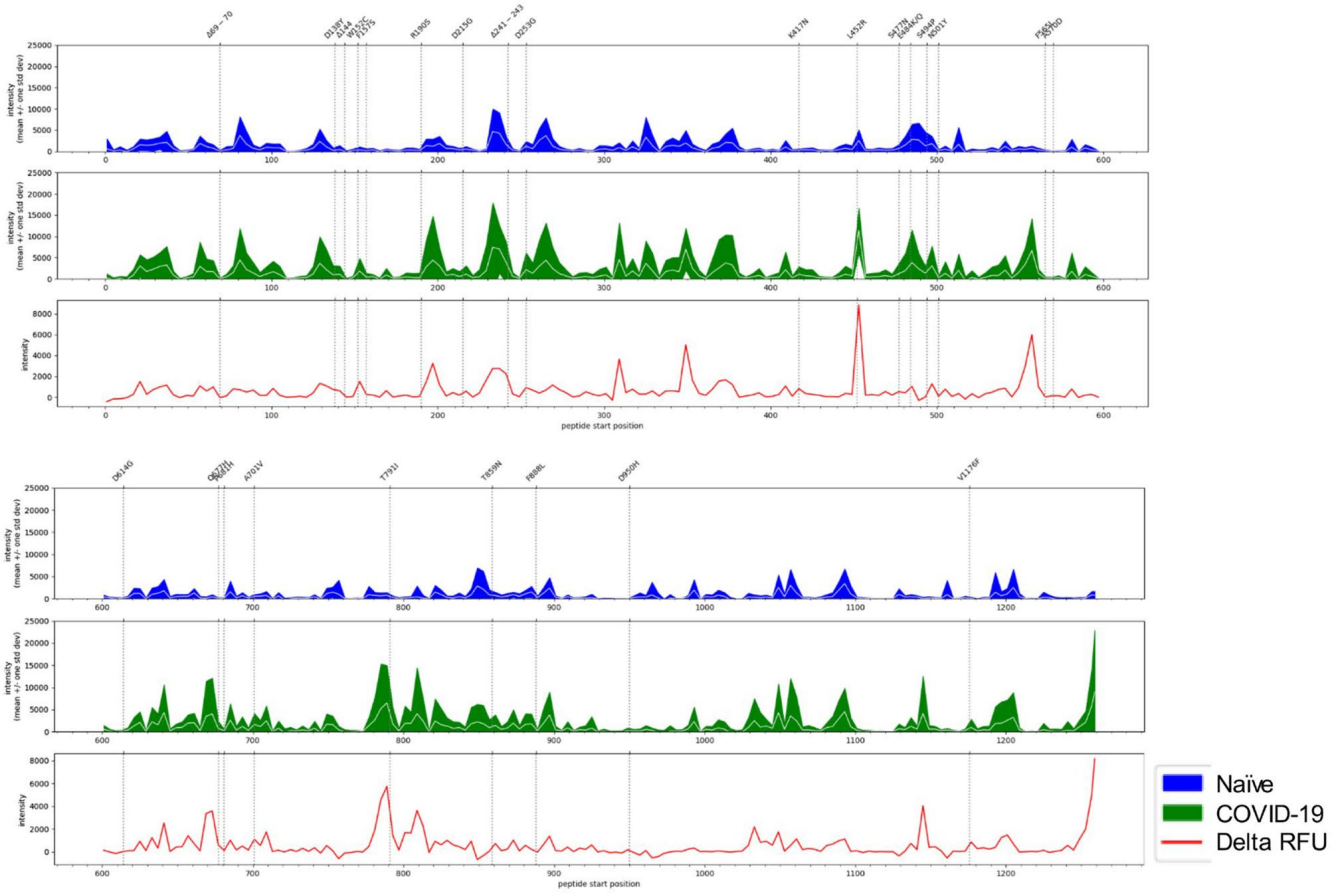
Spike protein immunosignature reveals long-term antibodies bind key regions associated with emerging variants of concern. X-axis represents the linear protein sequence arranged N-terminus (left) to C-terminus (right). The top trio of panels represent positions 0 to 600, the bottom trio of panels represent positions 600–1273. The white line in the middle of the band represents the mean, while the width of the band represents ± 1 standard deviation. Values were generated using naïve serum samples (n = 20, blue band), and COVID-19 sera (n = 22; green band). Immunosignature showing differential reactivity (red line, delta relative fluorescent unit [delta RFU]) was generated by comparing the mean signal intensity of naïve sera to that of COVID-19 sera. Polymorphisms were retrieved from the Center for Disease Control.

COVID-19 convalescent sera collected in this study were from individuals infected with what is almost certainly to the original Wuhan or D614G lineage of SARS-CoV-2. These cases occurred at the onset of the pandemic (March 2020) in Saskatchewan, Canada when the original Wuhan and D614G lineages were the only circulating variants in this province, and within the country, and during the time-frame of initial infection and blood collection^26^. We generated an immunosignature for SARS-CoV-2 spike protein (Fig. 6) to determine if IgG antibodies within COVID-19 convalescent sera bound to regions that coincided with the mutations found in the emerging variants of concern. We found that mutations L452R and L452Q neighbors spike epitope 453–467, one of the most differentially reactive peptides with COVID-19 convalescent sera. This mutation is associated with variants of concern including B.1.427 (Epsilon), B.1.429 (Epsilon) lineages, B.1.526.1 (Iota), B.1.617 (Delta and Kappa) lineages, and C.37 (lambda). In addition, elevated reactivity is observed at residues 485–499 with both COVID-19 and naïve sera, however this reactivity is not discriminatory with approximately 50% of samples in both cohorts showing strong reactivity to this region. Like L452R/Q, this region is also within the RBD domain and contains key mutations (E484K, E484Q, F490S, and S494P) found in a number of emerging variants of concern including B.1.525 (Eta), P.2 (Zeta), P.1 (Gamma), B.1.351 (Beta), and C.37 (Lambda). These observations warrant further studies to address if long-term immune pressure at these specific sites may have been a contributing factor to these emerging mutations.

## Discussion

In the current study, we utilized peptide immunoarrays representing the proteome of SARS-CoV-2 to enable an unbiased approach in identifying long-term polyclonal, epitope-specific antibody responses to SARS-CoV-2 infection. When compared to similar single epitope level analyses completed at earlier time-points post-infection (< 60 days), we identified a smaller set of differentially reactive epitopes. Given that these serum samples contain antibodies that block RBD-ACE2 binding and/or neutralize live virus, these data point towards a much smaller subset of SARS-CoV-2 epitopes to further characterize for their contribution to antibody-mediated immunity. This smaller subset provides an advantage as the majority of spike-specific antibodies generated following infection are non-neutralizing^27,28^. Although epitopes beyond spike glycoprotein do not confer antibody-mediated protection, they represent an opportunity to refine serological-based diagnostic tests to aid in pathogen surveillance. The epitope-specific responses identified in the current study might demonstrate either durable antibody responses arising from long-lived plasma cells, or an evolving set of antibody responses arising from the on-going maturation occurring in germinal centers. These data provide further insight into the prolonged evolution and maintenance of the humoral immune response demonstrating that the state of circulating antibodies is dynamic, similar to the memory B cell pool, revealing the co-dominance of spike and VME1 at later time-points in contrast to spike and NCAP at earlier time-points. Additionally, these data, consistent with previous reports, reveal that the antibody response is heterogeneous, with few shared epitope-specific responses among COVID-19 cases. This finding has major consequences for evaluating humoral immunity given that the majority of antibodies induced by infection and vaccination are non-neutralizing and that responses to full-length spike does not capture the quality of the response. Measuring antibody responses towards linear epitopes associated with neutralizing targets adds another dimension to, and could significantly augment, how we currently evaluate the induction and duration of antibody responses to spike glycoprotein.

The cumulative reactivity of peptides within the S1 domain showed a weak positive correlation with S1 protein IgG titers measured by ELISA. This suggests that distinct epitopes are detected, likely a bias towards conformational (discontinuous) epitopes in the ELISA and linear (continuous) epitopes on peptide immunoarrays. This finding demonstrates that these complementary approaches provide a deeper understanding of the antibody responses to SARS-CoV-2. As importantly, as S1 antigen IgG titers weakly correlate with live virus neutralization titers, next-generation tests should consider the use of surrogate markers that capture functional antibody responses as the maintenance of protective immunity is the utmost concern with the ongoing pandemic.

Towards a complete understanding of antibody responses to SARS-CoV-2 also requires a high-resolution analysis to identify specific antigenic determinants. This information can guide the refinement of vaccine candidates exclusively towards neutralizing targets. Although the abundance of non-neutralizing antibodies with current COVID-19 vaccines^29^ has no negative consequences, restricting the induction of non-functional antibodies can mitigate potential adverse outcomes associated such as disease enhancement or interclonal B cell competition^30^ and might become relevant to the design of therapeutics for future coronavirus outbreaks or to specifically combat emerging variants. Lastly, the emergence and exponential increase of variants will require higher-resolution analyses to understand the interplay between host immunity, both naturally acquired and vaccine-induced, and the epitopes harboring these mutations. As the current peptide immunoarray was designed using sequence information derived from the original Wuhan virus, further work is warranted to determine if convalescent antibodies from these individuals similarly show a reduced binding capacity to linear epitopes harboring mutations present in each of the variants of concern.

Spike, NCAP, and VME1 epitopes identified in the current study are consistent with epitope-specific antibody responses reported previously^13–20^. This has generated a strong consensus regarding linear SARS-CoV-2 epitopes among different laboratories, geographically distinct populations, and platform technologies used to describe these responses (i.e., phage display, peptide ELISAs, and peptide immunoarrays). Although the sample population in the current study was small, the B cell epitopes identified strongly align with those identified among larger sample cohorts (n = 232^18^; n = 1051^16^). In the current study, we did not consider samples based on disease severity. In previous reports, epitope-specific antibody responses did not correlate with disease severity^16^ as observed with antibody responses to full-length spike glycoprotein or RBD^8^; only those that succumbed to infection were epitope-specific antibody responses significantly reduced^16^. Using phage display libraries, Shrock et al.^18^ showed that epitope-specific antibody responses in hospitalized COVID-19 patients were broader and greater in magnitude compared to non-hospitalized patients. Further study is needed to determine if disease severity affects the diversity of long-term epitope-specific antibodies following recovery.

In the current study, epitope-specific antibody responses were greatest for spike glycoprotein consistent with previous single epitope level analyses^13,17–19^ followed by VME1 and NCAP (Table 1). Similar numbers of differentially reactive NCAP and VME1 peptides were identified with COVID-19 convalescent sera collected at 5 months PD (Table 1), which contrasts with epitope-specific responses reported at earlier time-points (< 60 days) where NCAP was the immunodominant antigen and responses to VME1 were less frequently detected^13,17–19^. Further, epitope-specific responses to VME1 showed the highest response frequencies, suggesting B cell responses to this antigen have evolved and expanded by 5 months PD. For example, in the current study VME1 epitope 5–19 was highly antigenic among the COVID-19 convalescent serum samples tested with a responder frequency of 95%. By contrast, a lower responder frequency of 63% was previously reported^17^, and other reports have found that antibody responses to VME1 peptides early post-infection (< 60 days) were not frequently detected in COVID-19 convalescent sera^17–19^. In the current study, peptides in the C-terminus of VME1 were immunodominant epitopes consistent with previous reports^14^. However, a novel finding in this study was epitope-specific responses to epitope 145–159 which reacted with all COVID-19 convalescent serum samples. Taken together, these findings suggest that B cell responses to VME1 continue to expand following resolution of infection.

For nucleoprotein (NCAP), there is a consensus that two linear epitope clusters are frequently detected around residues 153–175 and 350–411^13,18,19^. The epitope cluster centered on residue 161 is highly reactive and has been proposed as a diagnostic peptide for SARS-CoV-2 infection^13,19^. However, among larger sample populations (n = 232), only one-third of samples reacted to NCAP peptides centered on this region^18^. The discrepancy in responder frequency could be a factor of the sample population size, or differences in the times at which antibody responses were measured post-infection (5–23 days vs 7 days). In the current study, NCAP peptide 161–175 had the highest signal intensity with a subset of COVID-19 convalescent serum samples; however, the response was highly variable resulting in a response frequency of 27%. We found the greatest responder frequency with NCAP peptide 233–247 (50%) and 393–407 (66%); the latter was previously reported to have the greatest reactivity with COVID-19 convalescent sera collected at 14 days post-infection^13^. Given that COVID-19 sera samples in the current study reacted with fewer NCAP peptides than that previously reported at earlier time-points (< 60 days) suggests a contraction of anti-NCAP antibodies circulating in blood at 5 months PD. Moreover, given that NCAP epitopes have been proposed as sero-diagnostic biomarkers, this study provides a better understanding of the durability of NCAP epitope-specific antibody responses, and identifies specific NCAP peptides that could increase the robustness of proposed peptide-based serological tests.

In the current study, we identified three unique antigenic regions in the RBD domain, and two unique epitopes in the RBM domain of spike glycoprotein. Identification of linear epitopes in the RBD and RBM of spike glycoprotein has been highly variable between studies. Some reports failed to identify immunodominant linear epitopes within the RBD domain or that the responder frequency to these linear epitopes was very low^13,16,17^*.* These observations have led to the conclusion that conformational epitopes are dominant in the RBD domain. However, given that these prior studies examined antibody responses in convalescent sera collected early post-infection (< 60 days), our data suggests that epitope-specific antibody responses to the RBD domain expand, and become more predominant, later post-infection. In support of this, longitudinal analyses of memory B cell repertoire has revealed that RBD-specific^9^ and spike-specific IgG^11^ clones expand over the course of 5–6 month following initial infection.

Within the RBD domain, linear B cell epitopes are consistently identified in four regions centered on the residues: 349, 417, 453, and 553^14–19^. These previous studies all examined epitope-specific antibody responses within 60 days post-infection. In the current study, convalescent sera collected at 5 months PD identified epitope-specific responses at each of these positions. The greatest responder frequency was observed with spike epitopes 453–467, 553–567, and 557–571; the latter having one of the highest response frequencies (68%) among the spike epitopes identified, and 453–467 displaying one of the greatest signal intensities. These data suggest that epitope-specific IgG responses to these peptides might represent more durable or long-lived antibody responses following infection. Localization of these peptide sequences on the 3D structure of spike glycoprotein trimer in the open conformation revealed surface accessible residues (Fig. 4). That these convalescent sera samples can neutralize live virus or block RBD-ACE2 binding suggests these circulating antibodies target critical regions within the RBD domain that can confer immediate protection upon re-infection. Co-crystallization of known neutralizing antibodies with spike glycoprotein has demonstrated that residues 452–460 and 470–474 are directly engaged in this interaction^16^. Moreover, epitope-specific depletion^20^, epitope blocking^17^, or epitope antibody enrichment^15^ experiments with COVID-19 convalescent sera and spike peptides consisting of residues 553–571 demonstrated the capacity of antibodies targeting this region to neutralize live virus or SARS-CoV-2 spike pseudovirus. Furthermore, spike epitopes 455–469 and 556–570 conjugated to virus-like particles induced IgG antibody responses in mice with neutralizing activity in pseudovirus neutralization assays^31^. Taken together with our observation that epitope 557–571 shows a stronger linear relationship with live virus neutralization titers than S1 IgG titers or RBD-ACE2 blocking lends further support that this epitope may be a more sensitive surrogate marker for evaluating antibody-mediated immunity, in addition to directly being a neutralizing target on spike glycoprotein.

There is a strong consensus among single epitope level analyses regarding the linear antigenic regions outside of the RBD domain. Linear epitopes in S1 N-terminal domain (NTD) domain have been identified in a few studies occurring early post-infection^13,19^. We observed a very low responder frequency (< 10%) for differentially reactive NTD peptides, with exception to 153–167 (responder frequency of 18%) suggesting that epitope-specific antibodies to this region are either mostly conformational or that linear epitope-specific antibodies rapidly contract following infection. Given that NTD-specific vaccine-induced antibodies are equally as effective at neutralizing live virus as those targeting RBD^29^ it warrants further attention to determine if antibodies against this region target linear and/or conformational epitopes in an effort to understand humoral immunity to coronavirus spike glycoprotein. Five regions are frequently detected using convalescent sera collected < 60 days post-infection. These regions are centered on residue(s): 570 (adjacent to the RBD domain), 660 (near the S1/S2 cleavage site), 760–800 (N-terminus of the S2 domain), 810 (S2’ cleavage site and FP1/FP2 domains), 1145 (HR2 domain), and 1256 (cytoplasmic tail). In the current study, we identified epitope-specific IgG responses localizing to these regions. In proximity to the S1/S2 cleavage site, we identified epitope-specific IgG antibodies to spike epitope 657–671, consistent with previous studies^13,16,17^. Centered on the N-terminus of the S2 domain we identified a cluster of epitopes in agreement with prior reports^13,16,18^. These epitopes are often associated with a high responder frequency, specifically those centered on residue 810 (45–80%); in the current study we observed a response frequency of 50% for spike epitope 805–819. Regarding the S2’ cleavage site and FP domains (residues 810–835), a number of previous analyses have found that this region is highly antigenic^13,14,16–20^. Moreover, epitope-specific antibody depletion of convalescent sera revealed that antibodies targeting this region contribute to virus neutralizing activity^20^, and in the mouse model epitope 793–812 conjugated to virus-like particles induced neutralizing antibodies^31^. A number of studies have determined that linear epitopes centered on 1145 are antigenic displaying a high responder frequency^13,15,17,18^; in the current study, the response frequency (45%) was lower than that previously reported. Epitope-specific enrichment of antibodies targeting this region neutralized viral activity in pseudovirus neutralization assays^15,17^. Recently, more direct evidence showed that human monoclonal antibodies specific to peptide 1148–1156 exhibited neutralizing activity against the live virus, and against vesicular stomatitis virus pseudotyped with SARS-CoV-2 spike variants and spike from related β-coronavirus^32^. This finding is consistent with the cross-reactivity of COVID-19 convalescent antibodies observed in the current study towards this peptide and those sharing sequence similarities from SARS-CoV, MERS-CoV, and hCoV-OC43 (Supplementary Fig. S2). Taken together, this lends further support that antibodies towards cross-reactive linear epitopes can provide broad-spectrum protection against β-coronaviruses^32^. Lastly, a cluster of epitopes localized to the cytoplasmic domain as observed previously^13,15,16^. This cluster had a much higher responder frequency in our samples (45–59%) then previously reported in convalescent sera samples collected < 60 days post-infection suggesting an expansion of these epitope-specific responses following convalescence. Collectively, linear epitope-specific responses are greater outside RBD and represent additional neutralizing target sites that could contribute to the development of next-generation multivalent vaccines with greater potential to target multiple variants and even more broadly protect against multiple β-coronaviruses.

Taken together, these data suggest that the circulating antibody repertoire for RBD-specific linear epitopes is restricted and that linear B-cell epitopes are most abundant outside the RBD domain. Antibody depletion, blocking, and enrichment experiments lend further support that these linear epitopes, beyond serving as surrogate markers, might directly contribute to functional antibody responses. These epitope-specific responses may serve as the front-line defense providing rapid serological immunity upon re-exposure, supported by the reactivation of the accumulating spike- and RBD-specific memory B cells^9,11,33^. An important question remains as to the extent that these antibody populations expand upon pathogen re-exposure, and which epitope-specific responses emerge from the memory B cell repertoire.

In summary, this study provides novel insight into the long-term, epitope-specific IgG responses in humans following resolution of SARS-CoV-2 infection at 5 months PD. These data provide a deeper understanding of the maintenance of functional antibody responses to SARS-CoV-2 at the single epitope level, and identifies significant changes occurring among the circulating antibody repertoire beyond the previous time-points (3–60 days). Advancing our knowledge of the long-term antibody-mediated immunity to SARS-CoV-2 is critical for understanding the evolution and kinetics of naturally acquired immunity to this novel coronavirus. Identifying these epitope-specific responses has implications for the pathogen biomarkers used for disease surveillance, defining surrogate markers of protective immunity, and can help refine the next-generation of β-coronavirus vaccines towards those that elicit antibody responses towards a wider range of neutralizing target sites.

## Methods

### Ethics statement

This project, and protocols used to collect human samples, received scientific and ethical approval from the Biomedical Research Ethics Board (Application ID 2387), University of Saskatchewan. All research was performed in accordance with the *Tri-Council Policy Statement: Ethical Conduct for Research Involving Humans Second Edition (December 2018)* and the *University of Saskatchewan Policies and Procedures for Ethics in Human Research (June 2013)*. Written informed consent was obtained from all participants; formal written consent was obtained from a parent or legal guardian of a minor participating in the study and verbal assent was obtained from the participant.

### Study cohort

COVID-19 convalescent sera (n = 22; Supplementary Table S1) were kindly provided by the Roy Romanov Provincial Health Laboratory (Regina, Saskatchewan). COVID-19 patients were confirmed positive for SARS-CoV-2 by RT-PCR (Roy Romanov Provincial Health Laboratory) in March of 2020, and blood serum collected in July or August 2020. Naïve individuals (n = 37) were recruited to this study in November 2020. Inclusion criteria for the naïve individuals included no prior infection with SARS-CoV-2, no prior symptoms associated with COVID-19 in the last 6 months, and no contact with known or suspected COVID-19 cases. Serum was collected by blood venipunctures into serum-separator tubes. All individuals provided written informed consent, and samples de-identified.

### Peptide immunoarray assay

RepliTope™ Antigen Collection Pan-Coronavirus (Product Code: RT-HD-CoV2) microarrays were purchased from JPT Peptide Technologies (Berlin, Germany). Each array consists of 4416 unique peptides covering the full proteome of SARS-CoV-2, and spike glycoprotein, nucleoprotein, envelope small membrane protein, and membrane protein of SARS-CoV, MERS-CoV, and common cold coronaviruses HCoV-229E and HCoV-OC43. Each protein target is represented by consecutive 15-mer peptides with 11 amino acid overlap and printed in triplicate. All incubation steps were performed at room temperature on a rotating shaker. Peptide microarrays were blocked in Tris-buffered saline (TBS), pH 7.2 supplemented with 0.05% v/v Tween-20 (TBS-T) and 3% w/v bovine serum albumin fraction V (BSA; diluent) for 30 min. Serum was diluted 1:100 in diluent and incubated for 2 h. Each array was washed with 5 exchanges of TBS-T, and once with sterile deionized distilled water. Serum IgG antibodies were detected using Alexa Fluor 647 conjugated goat anti-human IgG, Fc(gamma) fragment specific antibody (Jackson ImmunoResearch, 109-605-098) diluted to 1 µg/mL in diluent and incubated for 45 min in the dark. Washes were carried out as previously described, and slides dried by centrifugation for 5 min at 800 × g.

### Peptide microarray imaging and analysis

Peptide microarrays were imaged using a GenePix Professional 4200A microarray scanner (MDS Analytical Technologies, Toronto ON, Canada) equipped with a 635 nm laser and fluorescence captured using a 655 to 695 nm filter. Images were scanned at 10 µm resolution and data acquired using GenePix software (version 6.0). Data was analyzed with the web-based service EPIphany (https://epiphany.usask.ca/epiphany/^34^). Briefly, background-corrected foreground was used to extract spot signal intensity and no normalization was applied. For each peptide, a Mann–Whitney U test was performed to determine if the distribution of intensities differed between COVID-19 and naïve groups, and *p* values were corrected for false-discovery using the Benjamini–Hochberg correction. A FDR-adjusted *p* value of 0.1 was considered statistically significant.

### S1-specific IgG ELISA

Recombinant 6xHistidine-tagged spike glycoprotein S1 domain (“S1 protein”) was expressed in HEK293T cells and purified by affinity chromatography. Immulon 2HB 96-well plates were coated with S1 protein (1 µg/mL) in sodium carbonate-bicarbonate, pH 9.6 buffer overnight at 4ºC. All subsequent steps were performed at room temperature. Each well was blocked with 5% non-fat skim milk powder in tris-buffered saline (TBS) containing 0.05% v/v Tween 20. Fourfold serial dilutions of serum in diluent (TBS containing 1% non-fat skim milk and 0.05% v/v Tween 20) starting at 1 in 100, in duplicate, were added to each well and incubated for 1 h. Secondary antibody [horseradish peroxidase (HRP) conjugated anti-human IgG (Jackson ImmunoResearch Inc.; 1 in 20,000 in diluent)] was added to each well and incubated for 1 h. Plates were washed with TBS containing 0.05% v/v Tween 20 following each incubation. HRP was reacted with OPD peroxidase substrate (0.5 mg/mL; Thermo Scientific Pierce 34006) for 30 min, the reaction stopped with 2.5 M sulfuric acid and the absorbance measured at 490 nm using a SpectraMax Plus 384™ Reader (Molecular Devices; USA). Antibody titers were determined using the reciprocal of the highest dilution that resulted in an absorbance value greater than the mean + 3 standard deviations (SD) of the absorbance value from serum samples obtained from negative controls (naïve sera).

### SARS-CoV-2 surrogate virus neutralization assay

To detect functional antibody responses in serum, the SARS-CoV-2 surrogate virus neutralization test kit was used (Cat. No. L00847, GenScript Inc., USA). The kit is an ELISA-based assay that mimics the virus neutralization process. The kit contains two key components, the HRP conjugated recombinant SARS-CoV-2 receptor binding domain (RBD) fragment (HRP-RBD) and the human angiotensin converting enzyme 2 (hACE2) receptor protein. The protein–protein interaction between HRP-RBD and hACE2 can be blocked by neutralizing antibodies that target the RBD domain of SARS-CoV-2 spike glycoprotein. The ELISA absorbance of the sample is inversely proportional to the titer of the anti-RBD neutralizing antibodies. The assay was performed using serum at a dilution of 1 in 10 using the provided diluent, and the results presented as percent inhibition as per the manufacturer’s instructions.

### Live virus microneutralization assay

All steps were performed in a Containment Level 3 facility. The virus was SARS-CoV-2/Canada/ON/VIDO-01/2020/Vero’76/p.2 (Seq. available at GISAID—EPI_ISL_425177). The virus was grown in Vero’76 (ATCC CRL-1587) cells. Virus microneutralization assays of the serum samples against SARS-CoV-2 virus were performed using the Vero’76 cell line. The serum samples were heat-inactivated for 30 min at 56 °C. Two-fold serial dilutions of serum were prepared in duplicate, starting at 1:20. Virus was added to the diluted serum and the mixture was incubated for 1 h at 37 °C in a humidified chamber with 5% CO_2_. Virus-serum mixtures (25 TCID50/well) were transferred to 96-well flat-bottom plates containing 90% confluent pre-seeded Vero’76 and incubated for 5 days in a 37 °C humidified chamber with 5% CO_2_. Microscopic evaluation was performed on day 1 post-infection to confirm no contamination, and evaluation on days 3 and 5 post-infection for cytopathic effects. The serum dilution factor for the last well with no cytopathic effects at 5 days post-infection was defined as the serum neutralization titer.

### Data and statistical analysis

Peptide microarray data analysis is described above (*Peptide Microarray Imaging and Analysis*). For this analysis, a FDR-adjusted *p* value less than 0.1 was considered statistically significant. Heat-maps and line graphs were generated using scripts written in python (V3.7.9) and utilizing the *heatmap* function from the Seaborn library (V0.11.1) and *pyplot.plot* function from the Matplotlib library (V3.3.3), respectively. Epitope maps and responder frequency graphs were generated using the ggplot2 (V3.3.3) and dplyr (V1.0.6) R packages. To calculate responder frequency, the mean signal intensity for all naïve sera samples (n = 20) for a given peptide was calculated and the cutoff set at 3 standard deviations above this value. Signal intensities in naïve and COVID-19 convalescent sera samples above this value were scored as a responder. Subsequent data analyses and visualizations were completed using GraphPad Prism (version 8; GraphPad Software, San Diego, California USA). To determine differences in the distribution of signal intensities between naïve and COVID-19 convalescent sera, a two-sample Kolmogorov–Smirnov test was used. Correlation analyses were performed using the Spearman rank-order correlation. To determine the diagnostic accuracy and performance of individual and multiple peptides, Receiver Operating Characteristic (ROC) curve analysis was performed and Area Under the Curve (AUC) calculated. Sequence alignments were performed using Clustal Omega^35^. PyMOL was used to display and color-code epitopes onto the 3D trimeric structure of spike glycoprotein retrieved from RCSB PDB (7DK3, SARS-CoV-2 S trimer, S-open^36^). *P* values less than 0.05 were considered statistically significant.

## Supplementary Information

Supplementary Information.

Supplementary Table S3.

## Data Availability

Data supporting the reported results is contained within the article and supplementary material. Peptide microarray data is available in the publicly accessible repository ArrayExpress under the accession number E-MTAB-10740.
