# Supplementary material for: High-resolution analysis of long-term serum antibodies in humans following convalescence of SARS-CoV-2 infection
Source: Sci Rep. 2022 May 31;12:9045. doi: 10.1038/s41598-022-12032-8 (PMC9152668; doi:10.1038/s41598-022-12032-8)
Supplement: Supplementary file 1 — Supplementary Information. [file 41598_2022_12032_MOESM1_ESM.pdf]

SUPPLEMENTAL INFORMATION

Supplementary Table S1. Patient demographics for COVID-19 convalescent sera collected 5 months post-diagnosis[a].

Sample ID	Sex	Age Range	DiaSorin Liaison Spike IgG[b,e]	S1 IgG Titer[e]	Surrogate virus neutralization[c]	Live virus neutralizing titer[d]
00003	Male	60-69	70.2	6106	93.5	224
00006	Male	60-69	101	25488	92.1	38
00007	Male	20-29	36.5	1388	55.0	10
00010	Female	50-59	53.3	1825	76.0	20
00012	Female	60-69	48.8	1793	80.8	20
00015	Female	30-39	16	441	47.4	0
00019	Male	15-20	23.8	417	49.5	0
00020	Female	70-79	71.6	2122	77.4	10
00021	Male	30-39	24	1401	78.7	0
00022	Male	40-49	22.6	1122	60.8	20
00023	Male	20-29	25.5	1167	69.1	0
00024	Female	20-29	34.8	1218	64.0	0
00025	Female	30-39	39.6	1434	63.2	10
00026	Female	40-49	66.2	4851	89.1	80
00028	Female	20-29	46.8	1623	80.0	40
00034	Female	60-69	68.6	5073	79.4	20
00035	Male	50-59	46.6	1649	76.3	56
00050	Male	30-39	22.3	1101	58.2	10
00054	Female	70-79	43.4	1387	74.3	40
00058	Female	30-39	20.4	1171	44.1	10
00059	Female	50-59	94.5	6663	96.9	112
00063	Male	20-29	37.4	1257	70.8	28

[a] RT-PCR positive patients from March 2020, and serum collected in July/August 2020.

[b] Detection of IgG against S1/S2 antigens of SARS-CoV-2. AU/mL; >=15, positive.

[c] Percent inhibition of recombinant SARS-CoV-2 RBD binding to human ACE2. Cutoff value of 30% or greater is interpreted as positive for SARS-CoV-2 neutralizing antibodies, as per the manufacturer.

[d] Microneutralization assay with live SARS-CoV-2 virus.

[e] Spearman rank correlation, r=0.948.

Supplementary Table S2. Patient demographics for serum samples collected from naïve individuals.

Sample ID	Sex	Age Range	S1 IgG Titer	Surrogate virus neutralization[a]
18	Female	20-29	80.50	4.68
26	Male	20-29	194	10.66
33	Female	20-29	0	7.69
34	Male	20-29	84.5	11.63
21	Male	30-39	22	7.83
22	Female	30-39	30	10.01
36	Male	30-39	0	13.11
37	Female	30-39	0	11.03
10	Male	40-49	0	0.05
13	Female	40-49	46.36	8.16
23	Female	40-49	39.5	9.45
24	Male	40-49	29.5	13.25
14	Male	50-59	0	8.67
16	Female	50-59	116.64	2.41
25	Male	50-59	54.5	4.17
27	Female	50-59	121	9.87
19	Male	60-69	0	5.79
20	Female	60-69	42.02	9.18
30	Male	60-69	27	9.78
32	Female	60-69	59	11.21

[a] Percent inhibition of recombinant SARS-CoV-2 RBD binding to human ACE2. Cutoff value less than 30% is interpreted as negative for SARS-CoV-2 neutralizing antibodies, as per the manufacturer.

Supplementary Table S3. Complete listing of peptides on the pan-coronavirus immunoarray and associated signal intensity recorded for each serum sample. [See MS Excel file].

Supplementary Table S4. Diagnostic specificity, sensitivity, and accuracy of SARS-CoV-2 immunodominant epitopes.

Antigen	Epitope position	Area Under the Curve	Specificity (Sp) and Sensitivity (Sn), [95% confidence interval]	Likelihood ratio
VME1	5-19	1.0<br>P < 0.0001	Sp=95 [76/99]<br>Sn=86 [85/100]	17.27
	145-159	1.0<br>P < 0.0001	Sp=95 [76/99]<br>Sn=100 [66/95]	20.00
NCAP	393-407	0.9682<br>P < 0.0001	Sp=95 [76/99]<br>Sn=77 [66/95]	15.45
Spike	453-467[a]	0.9614<br>P < 0.0001	Sp=95 [76/99]<br>Sn=77 [66/95]	17.27
	557-571[b]	0.9455<br>P < 0.0001	Sp=95 [76/99]<br>Sn=77 [56/89]	15.45
	789-803[c]	0.8977<br>P < 0.0001	Sp=95 [76/99]<br>Sn=77 [56/89]	15.45
	1253-1267[d]	0.8886<br>P < 0.0001	Sp=95 [76/99]<br>Sn=63 [42/80]	12.73
	a + b + c + d	0.979<br>P < 0.0001	Sp=95 [76/99]<br>Sn=81 [61/92]	16.36
	b + c + d + VME1 5-19 + VME1 145-159	1.0<br>P < 0.0001	Sp=95 [76/99]<br>Sn=100 [85/100]	20.00
Spike-S1 protein		1.0<br>P < 0.0001	Sp=95 [76/99]<br>Sn=100 [85/100]	20.00

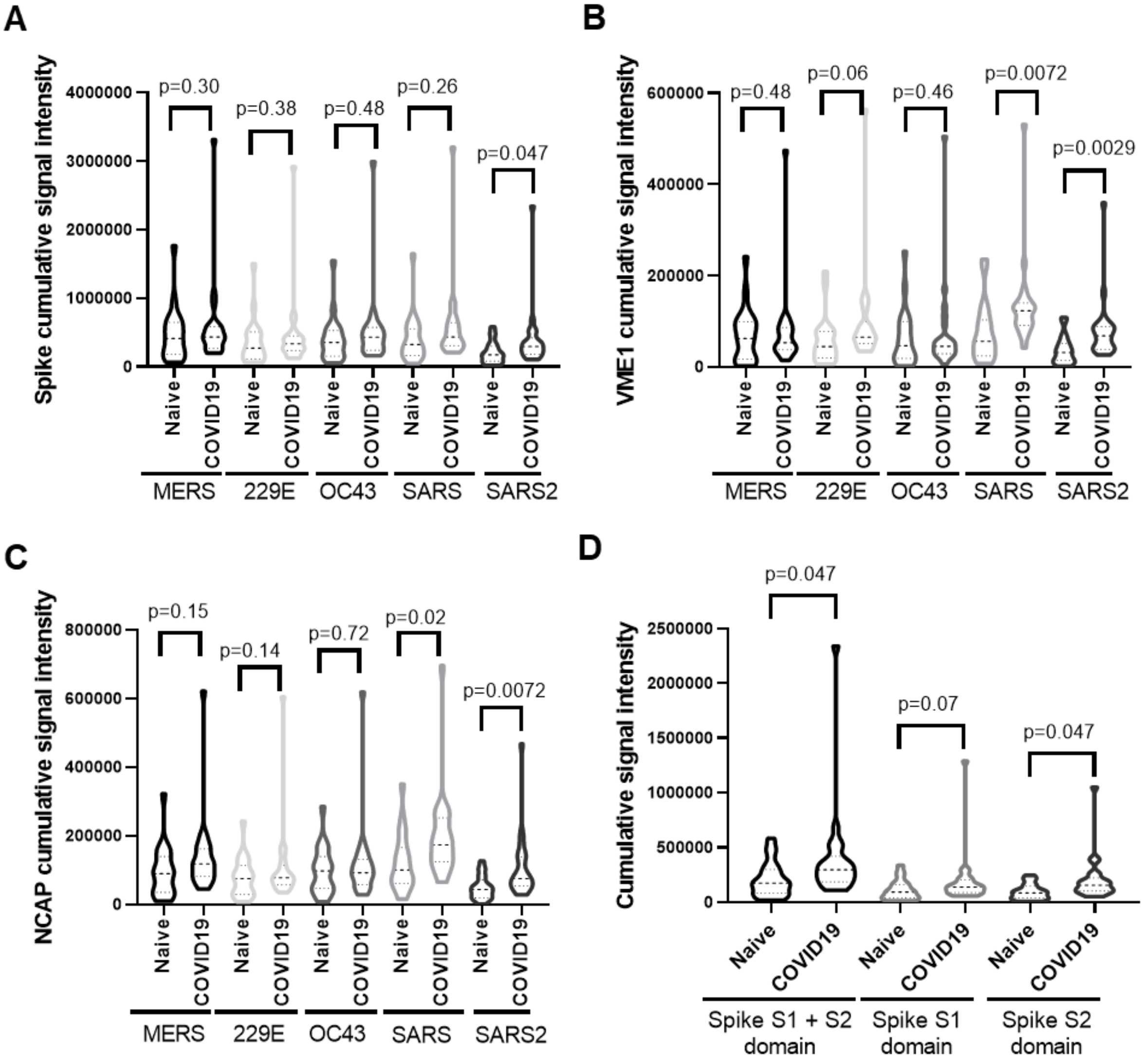

Supplementary Figure S1. Violin plots showing the distribution of reactivity among naïve (n=20) and COVID-19 convalescent sera (n=22) based on the sum of all peptide reactivities on the immunoarray. Reactivities restricted to (A) spike, (B) VME1 (membrane protein), and (C) NCAP (nucleoprotein). (D) Distribution of reactivity among the naïve (n=20) and COVID-19 convalescent sera (n=22) based on the sum of all peptide reactivities restricted to the complete SARS-CoV-2 spike protein (Spike S1 + S2 domain), Spike S1 domain, or Spike S2 domain. P values were calculated using a two-sample Komorgolorov-Smrinov test. Dashed line within the violin plot represents the median, dotted line represents the quartiles.

A

SPIKE MERS-CoV 885-899 SARSAIEDLLFDKVT
SPIKE HCoV-229E 685-699 RVAGRSAIEDILFSK
SPIKE HCoV-OC43 901-915 SSRSAIEDLLFDKVK
SPIKE SARS-CoV 789-803 PDPLKPTKRSFIEDL
SPIKE SARS-CoV-2 813-827 SKRSFIEDLLFNKVT

```
229E    N-----LSSVIPSLPTSGSRVAGRSAIEDILFSKLVTSGLGTVDADYKKCT--KGLSIAD 723
OC43    NVDDINFSPVLGCLGSECSKASSRSAIEDLLFDKVKLSDVGFVE-AYNNCT--GGAEIRD 936
MERS    N-----LTLLEP-VSISTGSRSARSAIEDLLFDKVTIADPGYMQ-GYDDCMQQGPASARD 922
SARS    N-----FSQILP-DP---LKPTKRSFIEDLLFNKVTLADAGFMK-QYGECL--GDINARD 830
SARS2   N-----FSQILP-DP---SKPSKRSFIEDLLFNKVTLADAGFIK-QYGDCL--GDIAARD 848
```

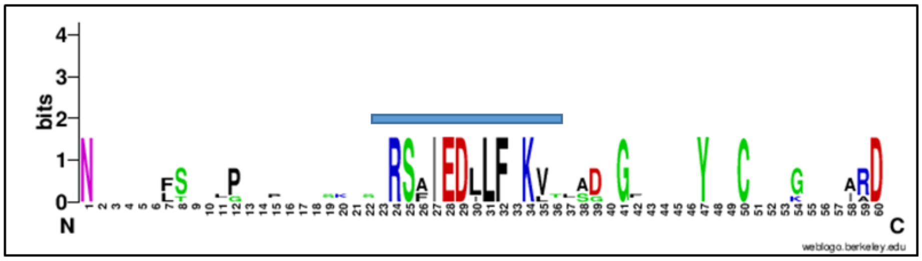

B

SPIKE MERS-CoV 1225-1239 NSTGIDFQDELDEFF
SPIKE HCoV-OC43 1229-1243 NLPDFKEELDQWFKN
SPIKE SARS-CoV 1125-1139 PELDSFKEELDKYFK
SPIKE SARS-CoV-2 1145-1159 LDSFKEELDKYFKNH

```
229E    NVTFVNISRSELQTI--VPEYIDVNKTLQELSYKLPNYTVPDL-VVEQYNQTILNLTSEI 1071
OC43    AVNYTKAPYVMLN--TSIPNLPDFKEELDQW-FKNQTSVAPDL-SLDYINVTF------- 1260
MERS    VTYQN-ISTNLPPPLLGNSTGIDFQDELDEF-FKNVSTSIPNFGSLTQINTTL------- 1259
SARS    DVVIGIINNTVYDPLQ--PELDSFKEELDKY-FKNHTSPDVDLGDISGINASV------- 1158
SARS2   DVVIGIVNNTVYDPLQ--PELDSFKEELDKY-FKNHTSPDVDLGDISGINASV------- 1176
```

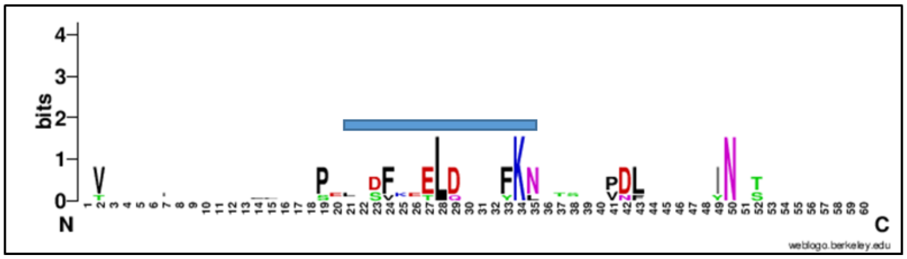

Supplementary Figure S2. **SARS-CoV-2 infection induces antibodies that cross-react with epitopes in related human coronaviruses.** Presented are the SARS-CoV-2 spike epitopes (A & B) that elicited cross-reactive antibody responses to peptides in related human coronaviruses. For each spike epitope: the top panel identifies the peptide printed on the immunoarray and the peptide coordinates within the linear sequence; the middle panel shows the sequence alignment with the printed peptide underlined; and the bottom panel shows the sequence logo generated from the sequence alignments displaying the frequency of specific amino acid residues at each position along the linear peptide sequence. The blue bar in the sequence logos identifies the SARS-CoV-2 epitope. Logos generated using WebLogo (Version 2.8.2; https://weblogo.berkeley.edu/logo.cgi).
